# Supplementary material for: Effectiveness of Colorectal Cancer Screening Promotion Using E-Media Decision Aids: A Systematic Review and Meta-Analysis
Source: Int J Environ Res Public Health. 2021 Aug 2;18(15):8190. doi: 10.3390/ijerph18158190 (PMC8345994; doi:10.3390/ijerph18158190)
Supplement: Supplementary file 1 [file ijerph-18-08190-s001.zip › ijerph-1291576-supplementary.pdf]

(“colorectal·cancer”·OR·“colon·cancer”·OR·“bowel·cancer”·OR·“rectal·cancer”·OR·“colorectal·neoplas*”·OR·“colorectal·carcinoma”)·AND·(“screening”·OR·“detection”·OR·“prevention”·OR·“colonoscopy”·OR·“fecal·occult·blood”·OR·“FOBT”)·AND·(“media”·OR·“mass·media”·OR·“electronic·media”·OR·“video”)¶

**Supplementary Figure S1.** Search strategy or the medical subject heading (MeSH) keywords used.

**Supplementary Table S1.** Risk of bias assessment according to the Cochrane's risk-of-bias tools for all articles in this review.

| Article ID | Randomization process | Deviations from intended interventions | Missing outcome data | Measurement of the outcome | Selection of the reported result | Overall Bias |
|---|---|---|---|---|---|---|
| Article 1[a] | Low | Low | Low | Low | Low | Low |
| Article 2[a] | Low | Low | Low | Low | Low | Low |
| Article 3[a] | Low | Low | Some concerns | Low | Some concerns | Some concerns |
| Article 4[b] | Low | Low | High risk | Some concerns | Some concerns | High risk |
| Article 5[a] | Low | Low | Low | Low | Low | Low |
| Article 6[a] | Low | Low | Some concerns | Low | Low | Some concerns |
| Article 7[a] | Low | Low | Some concerns | Low | Low | Some concerns |
| Article 8[a] | Low | Low | Low | Low | Low | Low |
| Article 9[a] | Low | Low | Low | Low | Low | Low |
| Article 10[a] | Low | Low | Low | Low | Low | Low |

[a]According to the risk-of-bias tool for randomized trials (RoB 2). [b]According to the risk-of-bias tool for cluster-randomized trials (RoB 2 CRT).

**Supplementary Table S2.** Risk of bias assessment according to domains using the Cochrane's risk-of-bias tool for randomized trials (RoB 2), and risk-of-bias tool for cluster-randomized trials (RoB 2 CRT).

| Adhering to intervention (the 'per-protocol' effect) | Randomization process | Deviations from intended interventions | Missing outcome data | Measurement of the outcome | Selection of the reported result | Overall Bias |
|---|---|---|---|---|---|---|
| Low risk | 10 | 10 | 6 | 9 | 8 | 6 |
| Some concerns | 0 | 0 | 3 | 1 | 2 | 3 |
| High risk | 0 | 0 | 1 | 0 | 0 | 1 |

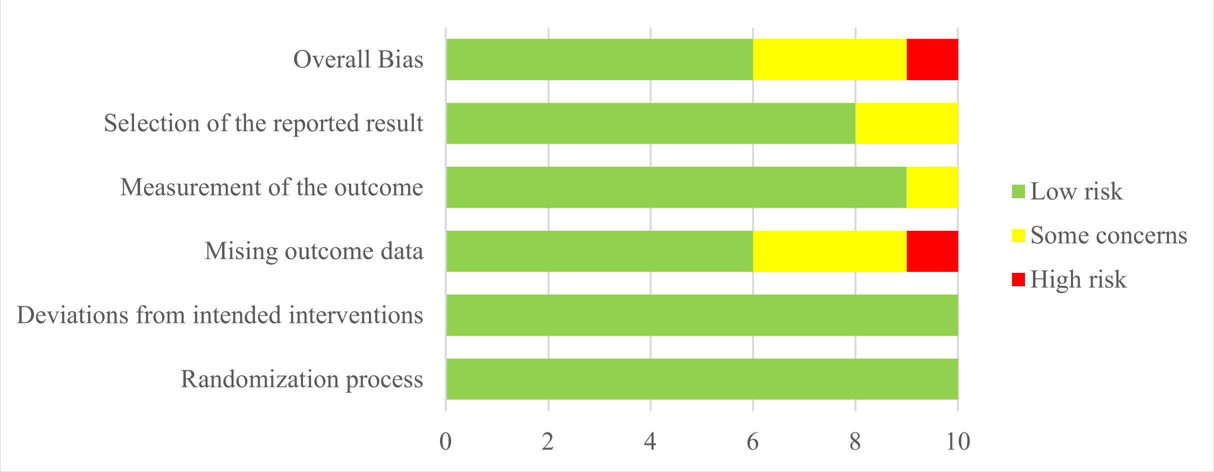


**Supplementary Figure S2.** Risk of bias assessment according to domains using the Cochrane's risk-of-bias tool for randomized trials (RoB 2), and risk-of-bias tool for cluster-randomized trials (RoB 2 CRT). Total number of study, N = 10.

**Supplementary Table S3.** Sources of funding for all articles in this review.

| Article ID | Source of Funding |
|---|---|
| Article 1 | Grant from the National Cancer Institute, USA (NIH-R01-CA-158027). |
| Article 2 | The New Zealand Ministry of Health with in-kind contributions from Waitematā District Health Board (12/NTA/91). |
| Article 3 | The Florida Department of Health's Biomedical Research Branch, Bankhead Coley (4BB09). |
| Article 4 | The vice-chancellor for Research and Technology, Hamadan University of Medical Sciences (No. 9309184585). |
| Article 5 | The American Cancer Society (RSG-13-165-01–CPPB). |
| Article 6 | Not stated. |
| Article 7 | Not stated. |
| Article 8 | The National Institutes of Health and National Cancer Institute, USA (R01CA115869). |
| Article 9 | The American Cancer Society (CCCDA-05-162-01). |
| Article 10 | The Centers for Disease Control and Prevention (CDC), USA (T01 CD000146). |
